# *Porphyromonas gingivalis* Promotes Glioma Progression via the PI3K/AKT/mTOR Signaling Pathway

**DOI:** 10.3390/cells15211998

**Published:** 2026-10-31

**Authors:** Mengyao Leng, Xiuhong Weng, Jingyi Luo, Chenxing Hou, Simin Wang, Zhiqiang Li, Bo Cheng

**Affiliations:** 1 Department of Stomatology, Zhongnan Hospital of Wuhan University, 169 Donghu Road, Wuhan 430071, China; lengmengyao@whu.edu.cn (M.L.); zn003825@whu.edu.cn (X.W.); luojingyi@whu.edu.cn (J.L.); 2023183030092@whu.edu.cn (C.H.); 2022283030209@whu.edu.cn (S.W.); 2 Department of Neurosurgery, Zhongnan Hospital of Wuhan University, 169 Donghu Road, Wuhan 430071, China

**Keywords:** glioma, *Porphyromonas gingivalis*, PI3K/AKT/mTOR pathway, tumor progression

## Abstract

**Highlights:**

1. The 16S rRNA and gingipain antigens of the periodontal pathogen *Porphyromonas gingivalis* (*P. gingivalis*) were detected in human glioma tissues.
2. *P. gingivalis* promoted glioma malignant progression by enhancing proliferation, mesenchymal transition and extracellular matrix remodeling.
3. *P. gingivalis* exerts its tumor-promoting effect through the PI3K/AKT/mTOR signaling pathway.
4. AKT inhibition and metronidazole attenuated *P. gingivalis*-induced glioma progression.

**What are the main findings?**

- Clinical and cellular evidence: The 16S rRNA signals and gingipains of *P. gingivalis* were detected in human glioma tissues, and *P. gingivalis* was shown to invade GBM cells, promoting proliferation, migration, and invasion through mesenchymal transition and ECM remodeling.
- Molecular mechanism and intervention: *P. gingivalis* promoted glioma progression through activation of the PI3K/AKT/mTOR pathway, while AKT inhibition effectively reversed these effects in vitro and in vivo.

**What are the implications of the main findings?**

- Biological implication: These findings highlight *P. gingivalis* as a previously unrecognized microbial factor capable of directly regulating glioma cell behavior and provide a mechanistic link between bacteria and oncogenic signaling.
- Therapeutic implication: AKT inhibition has therapeutic relevance in counteracting *P. gingivalis*-promoted glioma progression.

**Abstract:**

Glioma is the most prevalent and aggressive brain tumor with high recurrence rates and poor survival. *Porphyromonas gingivalis* (*P. gingivalis*), a major periodontal pathogen, has emerged as a potential contributor to neurological diseases. However, whether *P. gingivalis* is present in glioma tissues and directly promotes malignant progression remains unclear. Here, immunohistochemistry the detected gingipains in 11 of 13 human glioma specimens, while fluorescence in situ hybridization further detected *P. gingivalis*-targeted 16S rRNA signals in 3 of 13 specimens. These findings support the presence of *P. gingivalis*-associated signals in human glioma tissues. In cellular models, *P. gingivalis* invaded glioblastoma cells and promoted cell proliferation, migration, and invasion through induction of mesenchymal transition and extracellular matrix remodeling, thereby accelerating glioma progression. In a mouse xenograft model, *P. gingivalis* inoculation enhanced tumor growth and was associated with changes in proliferation, mesenchymal transition, and ECM remodeling-related markers. Mechanistically, *P. gingivalis* activated the PI3K/AKT/mTOR signaling pathway, while pharmacological AKT inhibition (AKTi-1/2) effectively suppressed these tumor-promoting effects. Metronidazole treatment also reduced tumor growth in vivo. Collectively, these findings identify *P. gingivalis* as a potential microbial contributor to glioma malignant progression and support further investigation of host–microbe interactions and AKT signaling as potential avenues for therapeutic research.

## 1. Introduction

Glioma is the most common primary malignant tumor of the central nervous system, accounting for 70% of adult malignant primary brain tumors [1]. Among all glioma subtypes, glioblastoma multiforme (GBM) is the most aggressive and lethal form, characterized by rapid proliferation, diffuse infiltration, and inevitable recurrence [2,3]. Despite advances in multimodal treatment strategies, the median overall survival of GBM patients remains only 14–16 months [4,5]. This poor prognosis is driven by multiple interconnected mechanisms [3]. Extensive cellular plasticity, epithelial–mesenchymal transition-like (EMT-like) changes, and extracellular matrix (ECM) remodeling collectively contribute to the highly invasive nature of GBM [6]. Moreover, the highly hypoxic and immunosuppressive microenvironment of GBM further facilitates tumor aggressiveness and therapeutic resistance [7]. These malignant phenotypes are orchestrated by multiple oncogenic signaling pathways, among which aberrant activation of the PI3K/AKT/mTOR pathway plays a crucial role [8,9].

Periodontitis is a chronic inflammatory disease, affecting nearly 62% of dentate adults [10]. *Porphyromonas gingivalis* (*P. gingivalis*, *P.g*) is a keystone periodontal pathogen closely associated with the progression and recurrence of periodontitis [11,12]. Increasing evidence also indicates that *P. gingivalis* can colonize tumor tissues and promote malignant progression in several cancers, such as oral squamous cell carcinoma (OSCC) [13], esophageal squamous cell carcinoma (ESCC) [14], lung cancer [15], and pancreatic cancer [16]. Remarkably, research on the oral-brain axis has drawn growing interest recently, focusing on the involvement of oral dysbiosis and periodontitis in multiple brain diseases, such as Alzheimer’s disease (AD) [17,18], Parkinson’s disease [19,20], depression [21,22], brain abscesses [23,24], and brain tumors [25]. *P. gingivalis* can cross the blood–brain barrier (BBB) or mediate neuroinflammation and injury through a variety of pathways, including the gut–brain axis, ultimately participating in the onset and progression of neurodegenerative diseases [26,27]. *P. gingivalis* and its virulence factor gingipain have been detected in the brains of Alzheimer’s disease and Parkinson patients [18,28], while oral-derived microbial signatures have also been identified in brain tumors [29]. Wen et al. [25] reported significant associations between oral microbial taxa and glioma grade. *P. gingivalis* gingipain antigens have been detected in GBM tissues and *P. gingivalis* infection was found to upregulate IL-6 and PD-L1 expression while enhancing the invasiveness of human GBM cells [30]. In addition, our previous study demonstrated that lipopolysaccharide (LPS) derived from *P. gingivalis* promotes the proliferation and migration of GBM cells through activation of AKT signaling in vitro, and that glioma patients exhibit a significantly higher prevalence of poor periodontal status compared with benign tumor patients and the general population, with poorer periodontal conditions correlating with higher Ki-67 labeling indices [31]. Nevertheless, direct evidence of *P. gingivalis* colonization in human glioma tissues and its functional role in driving malignant progression through specific signaling pathways remains lacking.

In the present study, we investigated the presence of *P. gingivalis* in human glioma tissues and explored its effects on glioma malignant behaviors both in vitro and in vivo, further dissecting the underlying molecular mechanism mediated by the PI3K/AKT/mTOR pathway. For in vitro studies, we selected U87 and U251, two well-characterized GBM cell lines with distinct genetic background. U87 was chosen for in vivo subcutaneous xenografts in BALB/c nude mice due to its reliable engraftment and well-documented growth kinetics; the immunodeficient phenotype permits human tumor cell survival and enables direct assessment of bacterial and drug effects on tumor growth without adaptive immune interference.

Our study establishes a novel link between *P. gingivalis* colonization and glioma progression, revealing a previously unrecognized microbial driver of tumor malignancy. These findings suggest a potential therapeutic target for glioma patients with *P. gingivalis* colonization.

## 2. Materials and Methods

### 2.1. Human Tissue Specimens

Tumor tissues were collected from 13 patients with glioma treated at the Zhongnan Hospital of Wuhan University (Wuhan). All patients were preoperatively diagnosed with glioma and underwent radical resection. Due to ethical reasons, non-tumor brain tissues as controls were absent. This study was approved by the Ethics Committee of Zhongnan Hospital of Wuhan University (NO. 2021043) and conducted according to the tenets of the Declaration of Helsinki. Signed informed consent was obtained from all participants. Demographic information, including age, sex (self-report), as well as clinical and sample-related information collected from medical records, are reported in Table 1.

### 2.2. Histological and Immunohistochemical (IHC) Staining

The tissues were fixed in 4% paraformaldehyde, subsequently dehydrated, cleared, embedded in paraffin, and finally sectioned into 4 μm slices. Hematoxylin and eosin (H&E) staining was performed on the sections following standard protocols. For IHC staining, antigen retrieval was conducted after deparaffinization and rehydration. Endogenous peroxidase activity was blocked with 3% H_2_O_2_, and sections were blocked with 5% goat serum. Primary antibodies against *P. gingivalis* (anti-Gingipain/*P. gingivalis*, Cat no. 61BG1.3, Developmental Studies Hybridoma Bank, DSHB, The University of Iowa, Iowa City, IA, USA), Ki67 (1:200, Cat no. #ER1706-46, Huabio, Hangzhou, China), MMP2 (1:200, Cat no. #R24993, Zenbio, Chengdu, China), and MMP9 (1:200, Cat no. #R380831, Zenbio, Chengdu, China) were incubated overnight at 4 °C, followed by biotinylated secondary antibody incubation and DAB color development. Sections were counterstained with hematoxylin and observed under a light microscope.

### 2.3. FISH Assay

A Cy3-labeled 16S rRNA probe specific for *P. gingivalis* (5′-Cy3-CTTGTAGTTCCGCCTACCTC-Cy3-3′; PG-POGI FISH Probe-CY3, catalog no. FBFP1340, F-Biology, Beijing, China) was used to detect bacterial localization in formalin-fixed, paraffin-embedded glioma tissue sections [32]. This probe sequence is well-established and previously validated specifically targeting *P. gingivalis* 16S rRNA, with no reported cross-reactivity against closely related species. FISH detection was performed using a commercial kit (Gimma Gene, Shanghai, China) following the manufacturer’s standard protocols. After hybridization and washing, cell nuclei were counterstained with DAPI (C1006, Beyotime, Shanghai, China). Tissue sections were observed and imaged under a fluorescence microscope (Olympus, Hachioji, Tokyo, Japan). To define a positive sample, stringent morphological criteria were applied. A specimen was scored as positive only when distinct fluorescent signals exhibiting the typical bacterial morphology were observed against a clear, clean background. The final evaluation was independently performed by two pathologists in a double-blinded manner.

### 2.4. Culture of GBM Cell Lines

Human GBM cell lines (U87 and U251) were obtained from the American Type Culture Collection (ATCC). The cells were cultured in high-glucose Dulbecco’s Modified Eagle Medium (DMEM) supplemented with 10% fetal bovine serum, and maintained in a humidified incubator with 5% CO_2_ at 37 °C.

### 2.5. Culture of P. gingivalis and Cell Infection

*P. gingivalis* strain (*P.g*, ATCC 33277) was cultured according to standard anaerobic culture protocols. The *P. gingivalis* was cultured on brain heart infusion (BHI) broth agar plates containing 40% BHI, 10% agar, 0.5% hemin, 0.1% vitamin K1, and 20% defibrillated sheep blood under anaerobic conditions.

GBM cells were cultured to 90–95% confluence before bacterial infection. *P. gingivalis* was harvested at mid-logarithmic growth phase (OD600 ≈ 0.4), washed three times with pre-cooled PBS, and resuspended in PBS. Cells were infected with *P. gingivalis* at a multiplicity of infection (MOI) of 10 and incubated for 24 h. Uninfected cells were set as the blank control group for all subsequent assays.

### 2.6. Carboxyfluorescein Diacetate Succinimidyl Ester (CFSE)-Labeled P. gingivalis and Fluorescence Imaging

Bacterial pellets were resuspended in PBS and incubated with 10 μM CFSE (A1148024, AmBeed, Shanghai, China) at 37 °C for 30 min for fluorescence labeling. Labeled bacteria were washed twice with PBS and resuspended in cell culture medium prior to use. GBM cells were seeded in 24-well plates (2 × 10^4^ cells/well) and co-cultured with CFSE-labeled *P. gingivalis* for 24 h. After removal of the medium, cells were washed twice with PBS and stained with cell membrane red fluorescence staining reagent (DIL, C1991S, Beyotime, Shanghai, China) for 5 min at 37 °C in the dark. Following staining solution removal, cells were washed again with PBS, and nuclei were counterstained with DAPI (C1006, Beyotime, Shanghai, China). Images were acquired under a fluorescence microscope.

### 2.7. Sample Preparation for Transmission Electron Microscopy (TEM)

Cells were fixed with 2.5% glutaraldehyde, gently scraped and collected by centrifugation. Cell pellets were re-fixed, dehydrated with gradient ethanol, embedded in Epon-Araldite resin, sliced and stained with uranyl acetate and lead citrate. Ultrastructural characteristics of intracellular *P. gingivalis* in GBM cells were observed and photographed using a TEM (HT7800; Hitachi High-Tech Corporation, Tokyo, Japan).

### 2.8. Cell Counting Kit-8 (CCK-8) Assay

Cells were seeded in 96-well plates at a density of 3000 cells per well and incubated overnight for adherence. After treatment for 24, 48, and 72 h, the medium was replaced with 100 µL DMEM containing 10 µL CCK-8 reagent (C0041, Beyotime, Shanghai, China), and incubated at 37 °C for 1.5 h in the dark. The absorbance value at 450 nm was measured using a microplate spectrophotometer (Bio-Rad, Laboratories, Inc., Hercules, CA, USA).

### 2.9. 5-Ethynyl-2′-deoxyuridine (EdU) Assay

Cell DNA synthesis activity was detected using the BeyoClick™ EdU Cell Proliferation Kit with Alexa Fluor 594 (C0071S, Beyotime, Shanghai, China). Cells were incubated with EdU working solution for 2 h, fixed with 4% paraformaldehyde and permeabilized with 0.3% Triton X-100. Click reaction solution was added according to the kit instructions. After DAPI nuclear staining, EdU-positive cells were observed and quantified under an inverted fluorescence microscope.

### 2.10. Colony Formation Assay

Cells were seeded in 6-well plates at a density of 1000 cells/well and cultured for 2 weeks. Colonies were fixed with 4% (*v*/*v*) paraformaldehyde and stained with 0.1% (*w*/*v*) crystal violet. Colonies were photographed and statistically counted after PBS rinsing.

### 2.11. Cell Cycle Analysis

Cell cycle distribution was detected by flow cytometry using a Cell Cycle and Apoptosis Analysis Kit (C1052, Beyotime, Shanghai, China). After treatment, cells were harvested, fixed, and stained with propidium iodide according to the kit protocol. Cell cycle profiles were analyzed by flow cytometry and further quantified using FlowJo software (version 10.10; FlowJo LLC).

### 2.12. Wound-Healing Assay

Cells were seeded in 6-well plates and grown to 95% confluence. A linear scratch was made with a sterile 200 µL pipette tip. Cells were washed and cultured in serum-free medium. Wound closure was photographed at the indicated time points (0 h and subsequent intervals) to assess cell migration ability.

### 2.13. Migration and Invasion Assays

Cell migration and invasion abilities were assessed using 8-μm-pore Transwell chambers (Falcon^®^ 353097, Corning, Corning, NY, USA). For invasion assays, the upper chamber was pre-coated with Matrigel matrix (Corning, USA). GBM cells were resuspended in serum-free DMEM and seeded into the upper chamber, while medium containing 10% FBS was added to the lower chamber as a chemoattractant. After 24 h incubation, non-migrated cells on the upper membrane were wiped off. Migrated and invaded cells were fixed, stained with crystal violet, photographed and quantified.

### 2.14. Immunofluorescence (IF) Staining

GBM cells were seeded in 24-well plates and incubated with or without *P. gingivalis*. After treatment, cells were fixed in 4% (*v*/*v*) paraformaldehyde, permeabilized with 0.2% Triton X-100 and blocked with 5% BSA. Primary antibodies were incubated overnight at 4 °C, followed by incubation with fluorophore-conjugated secondary antibodies. Nuclei were stained with DAPI, and immunofluorescence signals were observed under a fluorescence microscope.

### 2.15. AKT Inhibition

Cells were assigned to four experimental groups: control (Ctrl), *P. gingivalis* alone, *P. gingivalis* plus AKTi-1/2, and AKTi-1/2 alone. Cells in the inhibitor groups were pretreated with 5 μM AKTi-1/2 (AKT inhibitor VIII, CAS No. 612847-09-3, MedChemExpress LLC, MCE, Monmouth Junction, NJ, USA) for 1 h before the indicated experimental procedures. All groups were processed under their respective conditions, and samples were collected for subsequent functional and molecular analyses.

### 2.16. In Vivo Subcutaneous Xenograft Model

All animal experimental procedures were approved by the Laboratory Animal Ethics Committee of Wuhan University (Approval No. ZN2022245 and date of approval 27 November 2022). Female BALB/c nude mice (4–5 weeks old) were purchased from WQJX Bio-Technology Co., Ltd. (Wuhan, China) and raised under specific pathogen-free conditions with free access to food and water. U87 cells (5.0 × 10^7^ cells/mL, 100 µL) were subcutaneously inoculated into the right flank of nude mouse. Tumor dimensions were measured every three days using a caliper throughout the experiment. Tumor volume was calculated using the formula: (V = (L\times W^2)/2), where (L) represents the tumor length and (W) represents the tumor width.

The in vivo study comprised two independent experiments. In the first experiment, mice were assigned to two groups (*n* = 5 per group; total *n* = 10). In the second experiment, mice were randomly assigned to four groups (n = 6 per = 5 group; total *n* = 24): (1) Ctrl, (2) *P.g*, (3) *P.g* + AKTi-1/2, and (4) *P.g* + MTZ. Group sizes were determined based on preliminary pilot experiments and previous studies using the same xenograft model. Mice were randomly assigned to treatment groups using a computer-generated random number table. Investigators performing tumor measurements and subsequent data analyses were blinded to group allocation.

Tumor-bearing mice received multipoint intratumoral injections of PBS or *P. gingivalis* (10^6^ CFU in PBS) every two days for 14 days to assess the effect of *P. gingivalis* on tumor growth. For intervention studies, mice were randomly assigned to four groups and treated for 14 days: (1) Ctrl: intratumoral PBS injection, intraperitoneal dimethyl sulfoxide (DMSO), and oral gavage of PBS; (2) *P.g*: intratumoral *P. gingivalis* injection, intraperitoneal DMSO, and oral PBS; (3) *P.g* + AKTi-1/2: intratumoral *P. gingivalis* injection, intraperitoneal AKTi-1/2 (dissolved in DMSO, 20 mg/kg/d), and oral PBS; (4) *P.g* + MTZ: intratumoral *P. gingivalis*, intraperitoneal DMSO, and oral MTZ (200 mg/kg/d).

Mice were monitored daily for body weight, activity, tumor appearance, and signs of distress. Humane endpoints were predefined as tumor diameter exceeding 15 mm, tumor ulceration or necrosis, or body weight loss exceeding 20% of baseline. No animals reached the predefined humane endpoints or were excluded from the analysis. On day 15 after treatment, all mice were euthanized by CO_2_ inhalation followed by cervical dislocation to ensure death. Tumors were harvested, weighed, and processed for histological and molecular analyses.

### 2.17. RNA Extraction and Quantitative Real-Time Reverse Transcription Polymerase Chain Reaction (qRT-PCR)

Total RNA was extracted using TRIzol reagent (Vazyme, Nanjing, China) and reverse transcribed into cDNA with the Prime Script^TM^ RT Master Mix (TaKaRa Bio Inc., Kusatsu, Shiga, Japan). qRT-PCR amplification of cDNA aliquots was performed using the SYBR^®^ Premix Ex Taq kit (TaKaRa, Japan) on a Step One Real-Time instrument (Thermo Fisher Scientific, Waltham, MA, USA). All steps were carried out according to the manufacturer’s instructions. Glyceraldehyde-3-phosphate dehydrogenase (GAPDH) was used as an internal control, and relative mRNA expression was calculated using the 2^−ΔΔCt^ method. Primer sequences are listed in Table 2.

### 2.18. Western Blotting (WB)

Total protein was extracted from GBM cells using RIPA lysis buffer (P0013B, Beyotime, Shanghai, China), and protein concentration was determined using the Bradford assay. Equal amounts of protein were separated by SDS–PAGE gels and transferred to PVDF membranes (Millipore, Merck KGaA, Darmstadt, Germany). Membranes were blocked with 5% nonfat milk and incubated overnight at 4 °C with primary antibodies, followed by goat anti-rabbit or anti-mouse IgG-horseradish peroxidase (HRP) (1:10,000, MCE, Monmouth Junction, NJ, USA) at room temperature for 1 h. Protein bands were visualized using an ECL kit (MCE, Monmouth Junction, NJ, USA), detected with the Chemiluminescence imaging system (Tanon), and quantified by densitometry with ImageJ software (version 1.54). β-Actin served as a loading control. The primary antibodies used are listed in Table 3.

### 2.19. Statistical Analysis

All experiments were performed in at least three independent replicates to ensure reproducibility and objectivity. All statistical analyses were conducted with GraphPad Prism 10.1.2 (USA). Data are presented as mean ± SD. Normality was assessed using the Shapiro–Wilk test. For comparisons between two groups, unpaired Student’s *t*-test was applied to normally distributed data, while the Mann–Whitney U test was used for non-normally distributed data. Multiple group comparisons were analyzed by one-way or two-way ANOVA as appropriate. *p* < 0.05 was considered as statistical significance. (* *p <* 0.05, ** *p <* 0.01, *** *p <* 0.001, and **** *p <* 0.0001).

### 2.20. Transcriptomic Analysis

RNA-seq was performed using the BGISEQ-500 platform (MGI Tech Co., Ltd., Shenzhen, China), with three independent biological replicates per group (*n* = 3), generating an average of 6.68 Gb of sequencing data per sample. Differentially expressed genes (DEGs) between control and *P. gingivalis*-infected cells were identified using the limma package (version 3.60.2) in R (version 4.4.1). Genes with |log2FC| ≥ 0.58 (equivalent to a fold change of ≥1.5 or ≤0.67) and a Benjamini–Hochberg-adjusted *p* value < 0.05 were considered differentially expressed. KEGG pathway enrichment analysis was performed using the clusterProfiler package (version 4.12.6), with Benjamini–Hochberg correction for multiple testing. Pathways with an adjusted *p* value < 0.05 were considered significantly enriched.

## 3. Results

### 3.1. P. gingivalis Is Detected in Human Glioma Tissues and Invades GBM Cells

**Figure 1:**
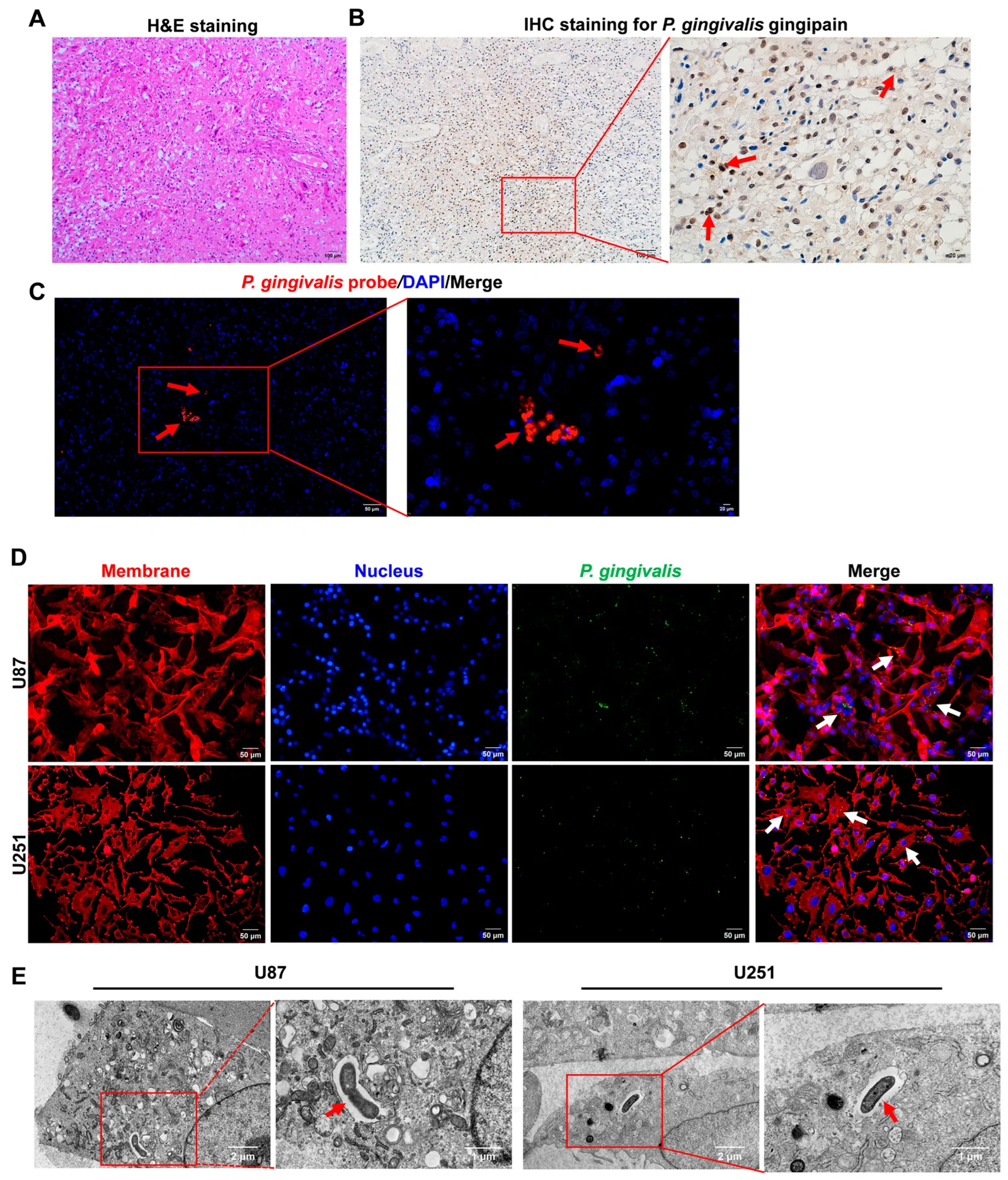
*P. gingivalis* is detected in human glioma tissues and invades GBM cells. (**A**) Representative H&E staining of human glioma tissue. Scale bar: 100 μm. (**B**) Representative immunohistochemical staining showing positive gingipain expression in glioma tissue. Scale bar: 100 μm and 20 μm. (**C**) Representative 16S rRNA fluorescence in situ hybridization (FISH) images of human glioma tissues. A Cy3-conjugated oligonucleotide probe (red) specific for *P. gingivalis* 16S rRNA was used. Nuclei were counterstained with DAPI (blue). Red arrows indicate positive signals. Scale bars: 50 μm and 20 μm. (**D**) Representative fluorescence images showing intracellular localization of CFSE-labeled *P. gingivalis* (green) in U87 and U251 GBM cells after 24 h of co-culture at a multiplicity of infection (MOI = 10). Cell membranes were stained with DiL (red), and nuclei were counterstained with DAPI (blue). Scale bar: 50 μm. White arrows indicate cells showing clearly visible intracellular green fluorescence signals, consistent with the presence of *P. gingivalis*. (**E**) Transmission electron microscopy (TEM) images of internalized *P. gingivalis* within the cytoplasm of U87 and U251 cells. The bacteria display intact rod-shaped morphology with a distinct double-layered cell wall (arrows). Scale bar: 2 μm and 1 μm.

To investigate the potential colonization of *P. gingivalis* in human glioma tissues, IHC analysis was performed on a cohort of glioma specimens, including GBM, using the specific antibody 61BG1.3, which recognizes the hemagglutinating proteases present on all tested strains of *P. gingivalis*, including RgpA (gingipain R1; also known as PrpR1 or hemagglutinin HagE), Kgp (lysine-gingipain), and HagA (hemagglutinin A). Abundant gingipain signals were detected in 11 out of 13 glioma samples examined. Specifically, the brown gingipain staining was granular and occasionally diffuse, predominantly localized to the cytoplasm and occasionally in the nucleus, with a notably higher perivascular distribution compared to other parenchymal regions (Figure 1B). To further determine whether the gingipain signals detected in human glioma tissues indeed reflected the presence of *P. gingivalis*, we performed FISH targeting *P. gingivalis*-specific 16S rRNA on consecutive tissue sections. Notably, discernible positive fluorescent signals were observed in 3 out of the 13 glioma specimens (Figure 1C). These results provide complementary evidence indicating the presence of *P. gingivalis*-associated signals in human glioma specimens.

Research has reported that *P. gingivalis* is an intracellular colonizing bacterium capable of invading, replicating in, and surviving in various cancer cells, and then exert pathogenic effects [33,34]. Therefore, we hypothesized that *P. gingivalis* could also invade and colonize within GBM cells. To test our hypothesis, human GBM cell lines U87 and U251 were cocultured with *P. gingivalis*. Fluorescence images showed a prominent perinuclear bacterial signal in both GBM cell lines following co-culture, indicating the intracellular localization of *P. gingivalis* (Figure 1D). TEM revealed rod-shaped bacteria with intact double-membrane structures enclosed within host-derived phagosomes in ultrathin sections of GBM cells. This morphological evidence further corroborates the intracellular localization of *P. gingivalis* within GBM cells (Figure 1E). Together, these observations provide evidence for the presence and intracellular localization of *P. gingivalis* in glioma tissues, revealing a previously unrecognized association between *P. gingivalis* and glioma.

### 3.2. P. gingivalis Promotes GBM Cells Proliferation In Vitro

**Figure 2:**
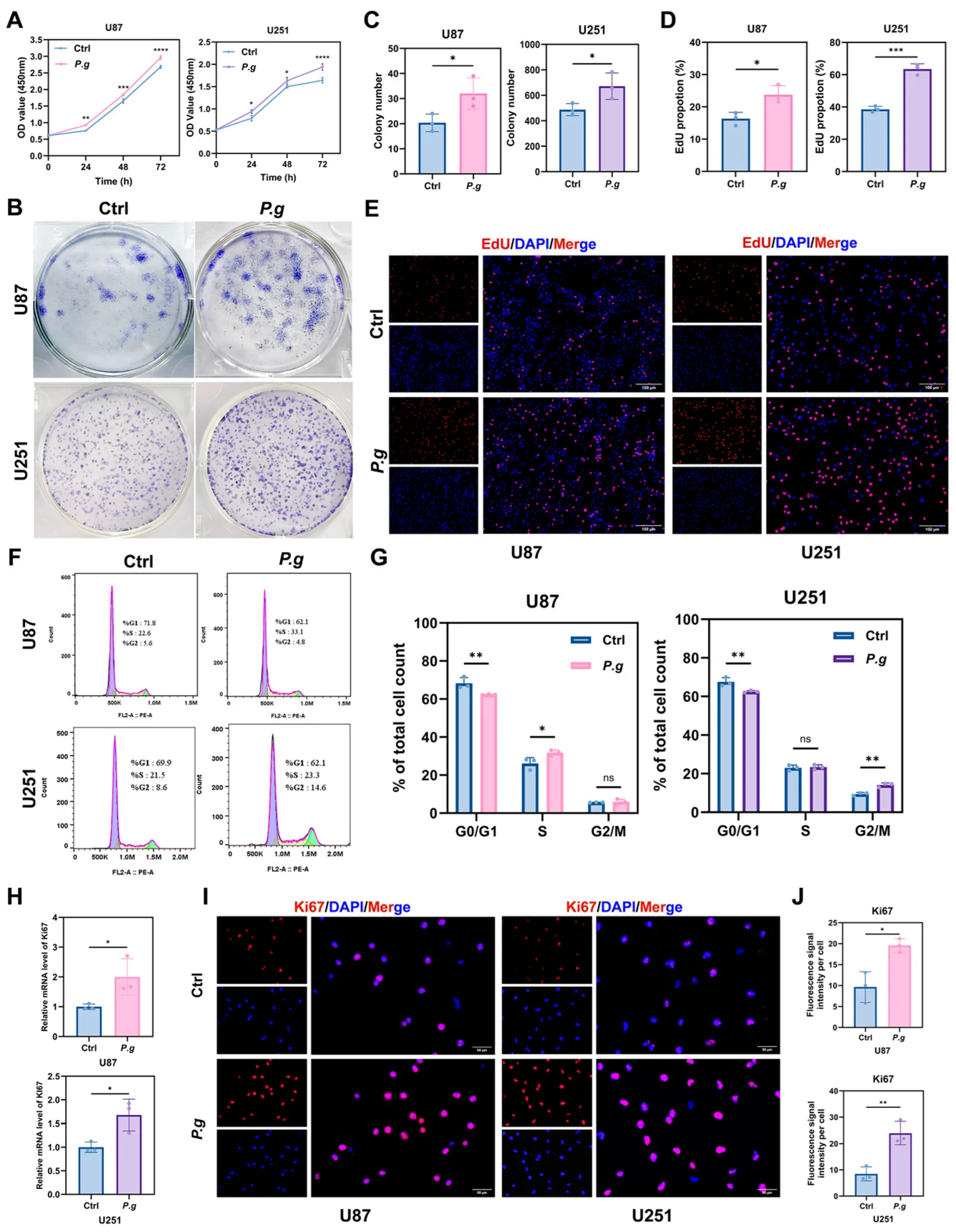
*P. gingivalis* promotes GBM cells proliferation in vitro. (**A**) CCK-8 assay assessing the proliferation abilities of U87 and U251 cells following treatment with PBS or *P. gingivalis* at 24, 48, and 72 h. Data were presented as mean ± SD (*n* = 3). (**B**) Colony formation assay of U87 and U251 cells treated with PBS or *P. gingivalis*. (**C**) Quantification of (**B**). Data were presented as mean ± SD (*n* = 3). (**D**) Quantification of EdU-positive cells. Data are expressed as percentage of EdU^+^ cells. Data were presented as mean ± SD (*n* = 3). Statistical analysis was performed using an unpaired two-tailed Student’s *t*-test. (**E**) Representative images of EdU incorporation assay in U87 and U251 cells treated with PBS or *P. gingivalis*. EdU-positive cells are shown in red, and Nuclei were stained with DAPI (blue). Scale bar: 100 μm. (**F**) Cell cycle analysis of U87 and U251 cells treated with PBS or *P. gingivalis* by flow cytometry. Representative histograms show DNA content distribution, with the percentages of cells in G1, S, and G2/M phases indicated. Shaded areas represent the respective cell cycle phases (G1, S, and G2/M), as determined using the Watson model. (**G**) Quantitative analysis of (**F**). Data were presented as mean ± SD (*n* = 3). (**H**) qRT-PCR analysis of Ki67 mRNA expression in U87 and U251 cells treated with PBS or *P. gingivalis*. Data were presented as mean ± SD (*n* = 3). (**I**) Representative immunofluorescence images of Ki-67 staining (red) in GBM cells with or without *P. gingivalis* treatment. Nuclei were counterstained with DAPI (blue). Purple/pink signals in the merged images indicate the colocalization of Ki-67 and DAPI signals. Scale bar: 50 μm. (**J**) Quantification of Ki-67 fluorescence intensity in GBM cells treated with or without *P. gingivalis*. Data were presented as mean ± SD (*n* = 3). Statistical analyses were performed using two-way ANOVA followed by Šídák’s multiple comparisons test for (**A**,**G**) and unpaired two-tailed Student’s *t*-tests for (**C**,**D**,**H**,**J**). Statistical significance: * *p* < 0.05, ** *p* < 0.01, *** *p* < 0.001, **** *p* < 0.0001; ns, not significant. Exact *p* values are provided in Supplementary Table S1.

Given that *P. gingivalis* has been shown to promote the proliferation of multiple cancer cells [16,35], we therefore investigated the impact of *P. gingivalis* on the proliferative capacity of GBM cells. The CCK-8 results showed that *P. gingivalis* can significantly facilitate GBM cells proliferation in a time-dependent manner (Figure 2A). Consistent with these findings, colony formation assays revealed that GBM cells co-cultured with *P. gingivalis* formed more total colonies compared with that observed when cells were cultured alone (Figure 2B,C). Through EdU incorporation assay, we found that co-culture with *P. gingivalis* led to a 47% and 52% increase in EdU-positive U87 and U251 cells, respectively, indicating enhanced DNA synthesis ability (Figure 2D,E). Furthermore, cell cycle analysis showed that *P. gingivalis* infection decreased the G0/G1-phase population (7.9% in U87; 9.6% in U251) while increasing the S and G2/M-phase populations in both cell lines (Figure 2F,G), indicating accelerated cell cycle progression. qRT-PCR and IF staining also confirmed that *P. gingivalis* stimulation significantly increased the expressions of Ki-67, a well-established proliferation marker (Figure 2H–J), further indicating enhanced cell cycle progression and DNA replication activity. Together, these results provide convergent evidence that *P. gingivalis* robustly promotes the proliferation of GBM cells.

### 3.3. P. gingivalis Accelerates GBM Cells Migration and Invasion via Mesenchymal Transition and ECM Remodeling

**Figure 3:**
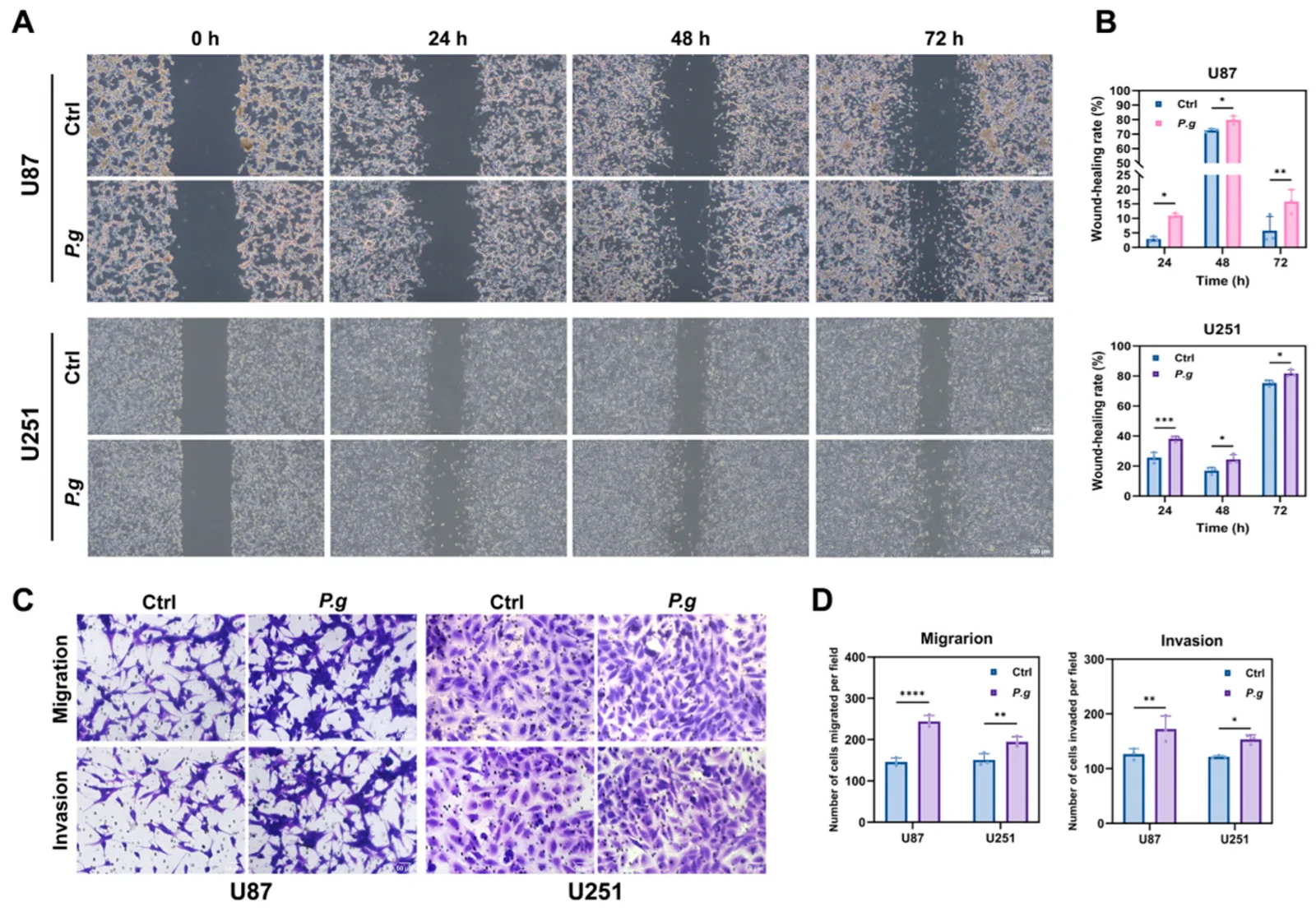
*P. gingivalis* accelerates GBM cells migration and invasion. (**A**) Representative images of wound healing assays in GBM cells treated with or without *P. gingivalis* at 0, 24, 48, and 72 h. (**B**) Quantitative analysis of wound closure. Data were presented as mean ± SD (*n* = 3). (**C**) Representative images of crystal violet-stained U87 and U251 cells that migrated or invaded through the membrane in Transwell assays following treatment with or without *P. gingivalis* are shown. (**D**) Quantification analysis of migrated or invaded cells. Data were presented as mean ± SD (*n* = 3). Statistical analyses were performed using two-way ANOVA followed by Šídák’s multiple comparisons test for (**B**,**D**). Statistical significance: * *p* < 0.05, ** *p* < 0.01, *** *p* < 0.001, **** *p* < 0.0001. Exact *p* values are provided in Supplementary Table S1.

High invasiveness represents another critical factor contributing to the malignancy and poor prognosis of glioma [36], which is primarily driven by mesenchymal transition and ECM remodeling [6]. We next explored the effects of *P. gingivalis* on the migration and invasion capabilities of GBM cells. Wound-healing assays showed that *P. gingivalis*-infected U87 and U251 cells exhibited notably higher wound closure rates at 24, 48 and 72 h compared to controls, with an increase of 74% (U87) and 33% (U251) at 24 h time point (Figure 3A,B). Transwell assays further confirmed that *P. gingivalis* significantly promoted both migration and invasion of U87 and U251 cells (Figure 3C,D).

**Figure 4:**
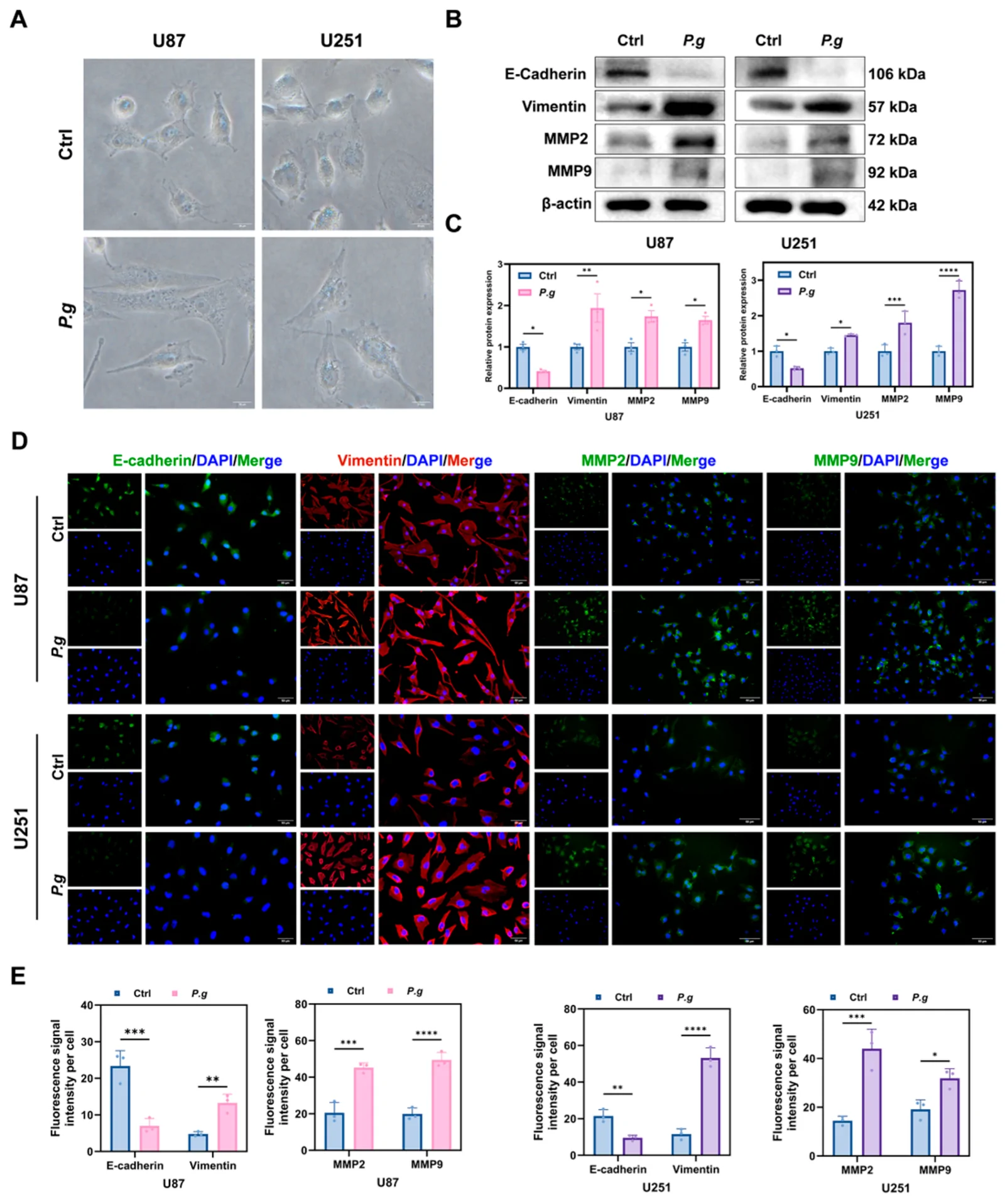
*P. gingivalis* induces mesenchymal transition and ECM remodeling of GBM cells. (**A**) Representative phase-contrast micrographs illustrating the morphological transition of U87 and U251 cells following *P. gingivalis* infection. (**B**) Western blotting (WB) analysis of migration- and invasion-related proteins (E-cadherin, Vimentin, MMP2, MMP9) in U87 and U251 cells treated with or without *P. gingivalis*. (**C**) Densitometric quantification of protein expression levels. Data were presented as mean ± SD (*n* = 3). (**D**) IF staining of E-cadherin (green), Vimentin (red), MMP2 (green) and MMP9 (green) in U87 and U251 cells treated with or without *P. gingivalis*. Nuclei were counterstained with DAPI (blue). Scale bar: 50 μm. (**E**) Quantitative analysis of immunofluorescence intensity. Data were presented as mean ± SD (*n* = 3). Statistical analyses were performed using two-way ANOVA followed by Šídák’s multiple comparisons test for (**C**,**E**). Statistical significance: * *p* < 0.05, ** *p* < 0.01, *** *p* < 0.001, **** *p* < 0.0001. Exact *p* values are provided in Supplementary Table S1.

Specifically, the *P. gingivalis*-infected cells underwent morphological transformation from a round shield shape with less pseudopods to an elongated and pseudopod-rich mesenchymal morphology (Figure 4A), which represents EMT-like characteristics [37]. Western blot and IF assays showed that *P. gingivalis* infection downregulated the epithelial marker E-cadherin while upregulated the mesenchymal marker Vimentin (Figure 4B–E), suggesting that *P. gingivalis* induces an EMT-like transition in GBM cells. In addition, *P. gingivalis* elevated the expression of MMP2 and MMP9 in GBM cells (Figure 4B–E). These molecular changes account for the enhanced migration and invasion capacities of *P. gingivalis*-infected GBM cells, as validated by wound healing and Transwell assays. Collectively, these results reveal that *P. gingivalis* promotes GBM cells migration and invasion by facilitating mesenchymal transition and ECM remodeling.

### 3.4. P. gingivalis Facilitates Glioma Growth In Vivo

**Figure 5:**
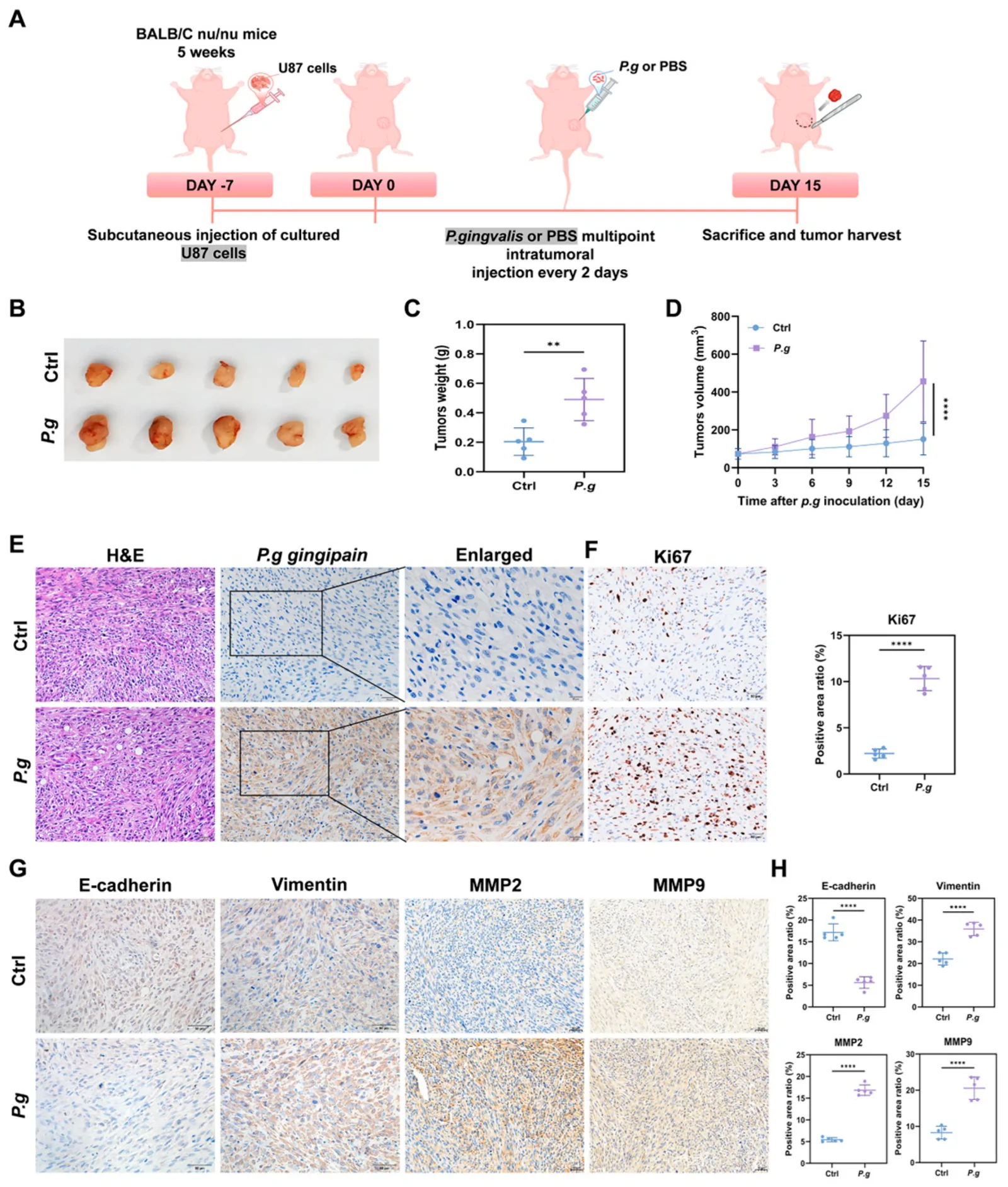
*P. gingivalis* facilitates glioma growth in vivo. (**A**) Schematic illustration of the subcutaneous xenograft model with intratumoral injection of *P. gingivalis* or PBS. (**B**–**D**) The macroscopic images of tumor tissues, weights and growth curves of subcutaneously xenografted tumor models in BALB/c nude mice following PBS or *P. gingivalis* infection. Data were presented as mean ± SD (n = 5 mice per group). (**E**) Representative images of H&E staining and immunohistochemical detection of *P. gingivalis* in tumor tissues. Scale bars: 50 μm and 25 μm. (**F**,**G**) Representative IHC staining images of Ki-67, E-cadherin, Vimentin, MMP2, and MMP9 in tumor tissues. Scale bars: 100 μm and 50 μm. Data were presented as mean ± SD (n = 5 mice per group). (**H**) Quantification of Ki-67, E-cadherin, Vimentin, MMP2, and MMP9-positive areas in tumor sections (n = 5 mice per group). Data are presented as mean ± SD. All statistical analyses in this figure were performed using unpaired two-tailed Student’s *t*-test. Statistical significance: ** *p* < 0.01, **** *p* < 0.0001. Exact *p* values are provided in Supplementary Table S1.

To assess the role of *P. gingivalis* in glioma growth in vivo, we established a xenograft model in nude mice (Figure 5A). Compared with control group, mice injected with *P. gingivalis* exhibited significantly accelerated tumor growth, as reflected by increased tumor weight and growth rate (Figure 5B–D). Notably, at the experimental endpoint, the *P. gingivalis* group showed a 139.7% increase in average tumor weight relative to the control group. IHC staining targeting gingipain demonstrated prominent immunoreactivity in mouse subcutaneous tumors derived from bacteria-inoculated animals, with no detectable signals in control tumors, supporting the presence of *P. gingivalis* in tumors from bacteria-inoculated mice (Figure 5E). In addition, IHC staining revealed that *P. gingivalis* infection upregulated the protein expression of Ki-67, Vimentin, MMP2, and MMP9, whereas E-cadherin expression was downregulated relative to control tumors (Figure 5F–H). These findings indicate that intratumoral *P. gingivalis* inoculation promotes tumor growth and is associated with changes in proliferation-, mesenchymal transition-, and ECM remodeling-related markers.

### 3.5. AKT Inhibition Attenuates P. gingivalis- Induced GBM Cells Malignant Progression

**Figure 6:**
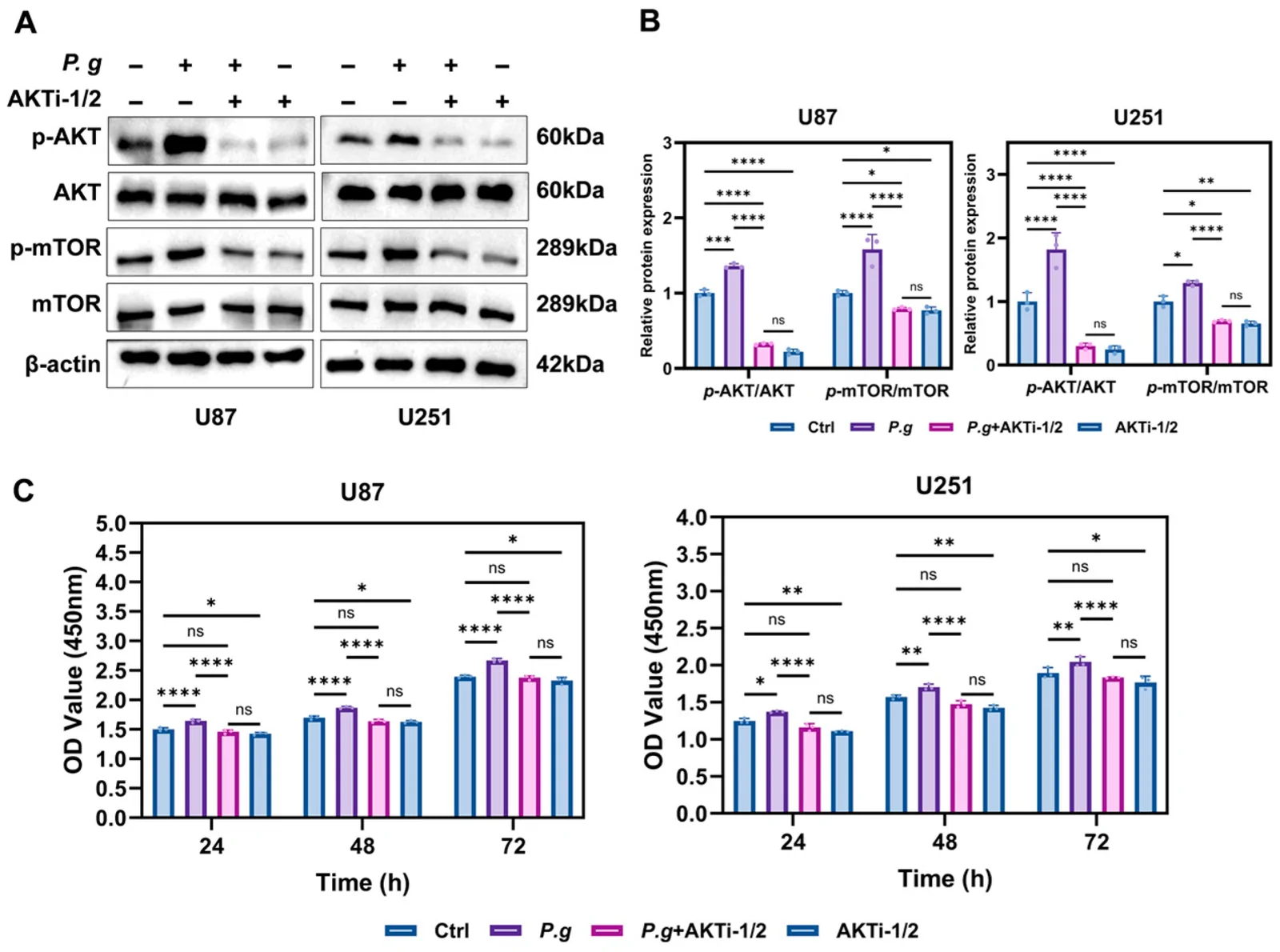
AKT inhibition attenuates *P. gingivalis*-induced GBM cells proliferation. (**A**) Representative Western blot images showing phosphorylated and total AKT and mTOR levels in the four experimental groups: control (Ctrl), *P. gingivalis* (*P.g*), *P. gingivalis* and AKTi-1/2 (*P.g* + AKTi-1/2), and AKTi-1/2 alone (AKTi-1/2). (**B**) Densitometric quantification of p-AKT/AKT and p-mTOR/mTOR ratios. Data were presented as mean ± SD (*n* = 3). (**C**) Cell proliferation assessed by CCK-8 assay. Bar graphs show the relative cell viability of U87 and U251 cells under indicated conditions: Ctrl, *P. g* infection (*P. gingivalis*), *P. gingivalis* and AKTi-1/2 (*P.g* + AKTi-1/2), and the AKTi-1/2-only group at specified time points. Data were presented as mean ± SD (*n* = 3). Statistical analyses were performed using two-way ANOVA followed by Šídák’s multiple comparisons test for (**B**,**C**). Statistical significance: * *p* < 0.05, ** *p* < 0.01, *** *p* < 0.001, and **** *p* < 0.0001; ns, not significant. Exact *p* values are provided in Supplementary Table S1.

Given the central role of the PI3K/AKT/mTOR pathway in glioma progression [38], and our previous observation that *P. gingivalis*-derived LPS activates this pathway [31], we hypothesized that live *P. gingivalis* may also activate this pathway in GBM cells. Transcriptomic profiling of *P. gingivalis*-infected GBM cells revealed the PI3K/AKT signaling pathway was significantly enriched in KEGG analysis (Figure S1A). Consistent with this finding, Western blot analysis showed increased phosphorylation levels of PI3K, AKT, and mTOR following *P. gingivalis* infection in GBM cells (Figure S1B,C). To investigate the contribution of AKT signaling to the observed phenotypic changes, we used the allosteric AKT inhibitor AKTi-1/2. As expected, AKTi-1/2 effectively suppressed *P. gingivalis*-induced AKT phosphorylation and subsequent mTOR activation (Figure 6A,B).

CCK-8 assays showed that while AKTi-1/2 treatment alone could suppress the proliferation of GBM cells compared to the control group, it significantly attenuated the enhanced proliferative capacity induced by *P. gingivalis* infection. Notably, the viability of the *P. gingivalis* + AKTi-1/2 group was markedly reduced compared to the *P. gingivalis* group, showing no statistically significant difference when compared to either the untreated control or the AKTi-1/2-alone group (Figure 6C). This phenomenon implies that AKT blockade could reverse the pro-proliferative effects triggered by *P. gingivalis* infection.

**Figure 7:**
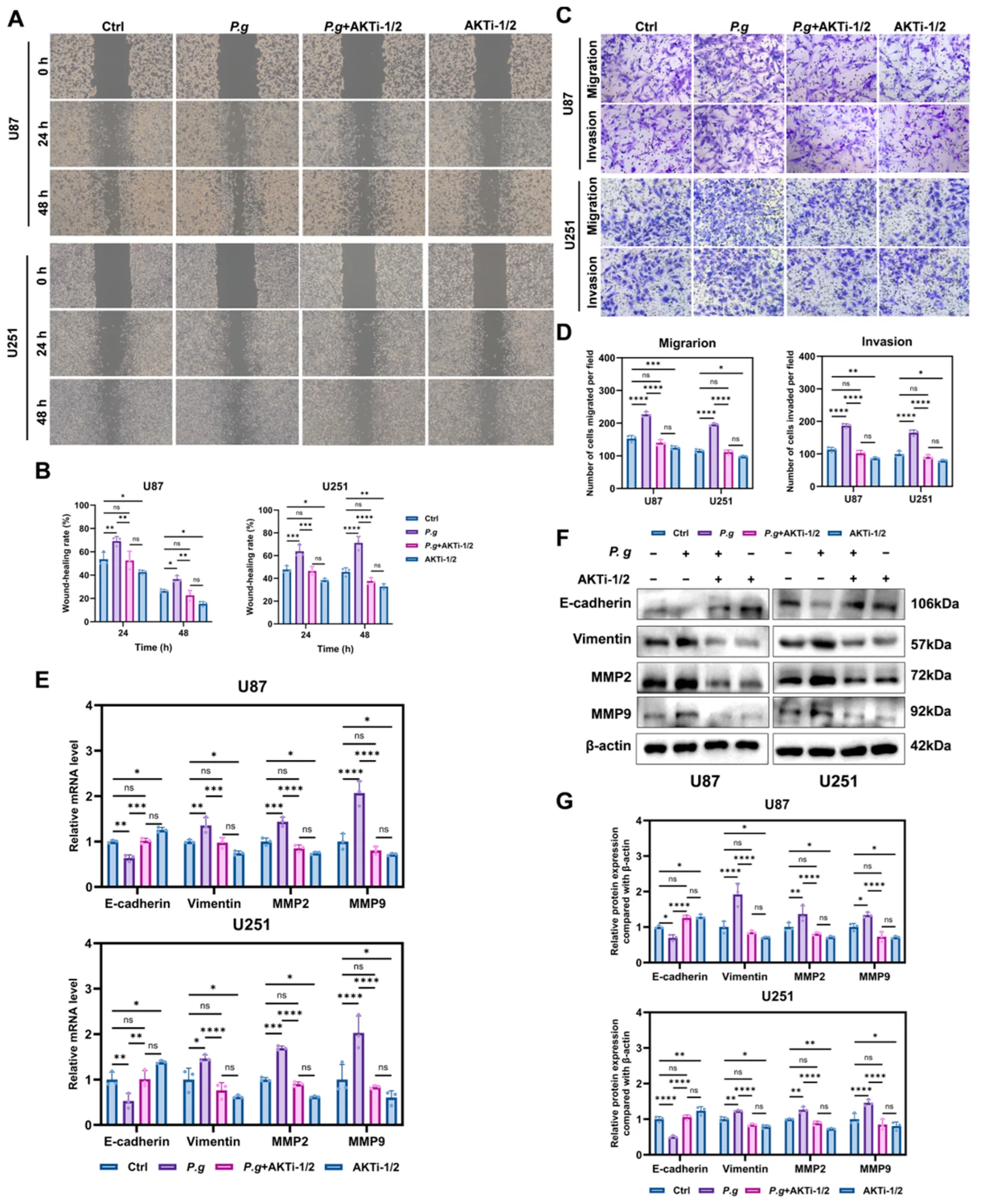
AKT inhibition attenuates *P. gingivalis*-induced migration and invasion in GBM cells. Cells were assigned to four groups: control (Ctrl), *P. gingivalis* (*P.g*), *P. gingivalis* and AKTi-1/2 (*P.g* + AKTi-1/2), and AKTi-1/2 alone (AKTi-1/2). (**A**) Representative images from wound-healing assay showing cell migration at 0, 24, and 48 h. Scale bar: 200 μm. (**B**) Quantitative analysis of wound-healing rates. Data were presented as mean ± SD (*n* = 3). (**C**) Representative images of Transwell migration and invasion assays. Scale bar: 50 μm. (**D**) Quantitative analysis of migrated and invaded cell numbers. Data were presented as mean ± SD (*n* = 3). (**E**) Relative mRNA expression levels of E-cadherin, Vimentin, MMP2, and MMP9 measured by qRT-PCR in the indicated groups. Data were presented as mean ± SD (*n* = 3). (**F**) Representative Western blot images showing the expression of E-cadherin, Vimentin, MMP2, and MMP9. (**G**) Quantification of protein expression levels normalized to loading control. Data were presented as mean ± SD (*n* = 3). All statistical analyses in this figure were performed using two-way ANOVA followed by Šídák’s multiple comparisons test. Statistical significance: * *p* < 0.05, ** *p* < 0.01, *** *p* < 0.001, **** *p* < 0.0001; ns, not significant. Exact *p* values are provided in Supplementary Table S1.

We next sought to determine whether this pathway supports a contribution of *P. gingivalis*-induced GBM cells migration and invasion. Wound-healing assays showed that *P. gingivalis* significantly accelerated GBM cells wound closure. Although AKTi-1/2 monotherapy inherently inhibited GBM cell migration compared to the control, AKTi-1/2 substantially abrogated the bacteria-induced hypermotility. Specifically, the migratory capacity in the combination group was significantly lower than that of the *P. gingivalis* group and showed no statistically significant difference compared to either the control or the AKTi-1/2-alone group (Figure 7A,B). Consistently, Transwell assays further demonstrated that AKTi-1/2 markedly reduced the increased migratory and invasive capacities of *P. gingivalis*-infected GBM cells. No statistically significant differences were detected between the *P.g* +AKTi-1/2 group and either the control or AKTi-1/2-alone group (Figure 7C,D).

To further elucidate the molecular basis underlying these phenotypic changes, we examined mesenchymal transition- and ECM remodeling-associated markers. Western blot and qRT-PCR analyses indicated that AKTi-1/2 significantly reversed the *P. gingivalis*-induced downregulation of E-cadherin and upregulation of Vimentin, MMP2, and MMP9, restoring their protein and mRNA expression to levels comparable to both the control and the AKTi-1/2-alone groups (Figure 7E–G). Together, these findings indicate that PI3K/AKT/mTOR axis may act as an essential downstream cascade whereby *P. gingivalis* facilitates mesenchymal transition and ECM remodeling, thereby enhancing the migratory and invasive potential of GBM cells.

### 3.6. AKTi-1/2 and MTZ Attenuate P. gingivalis-Induced Glioma Progression In Vivo

**Figure 8:**
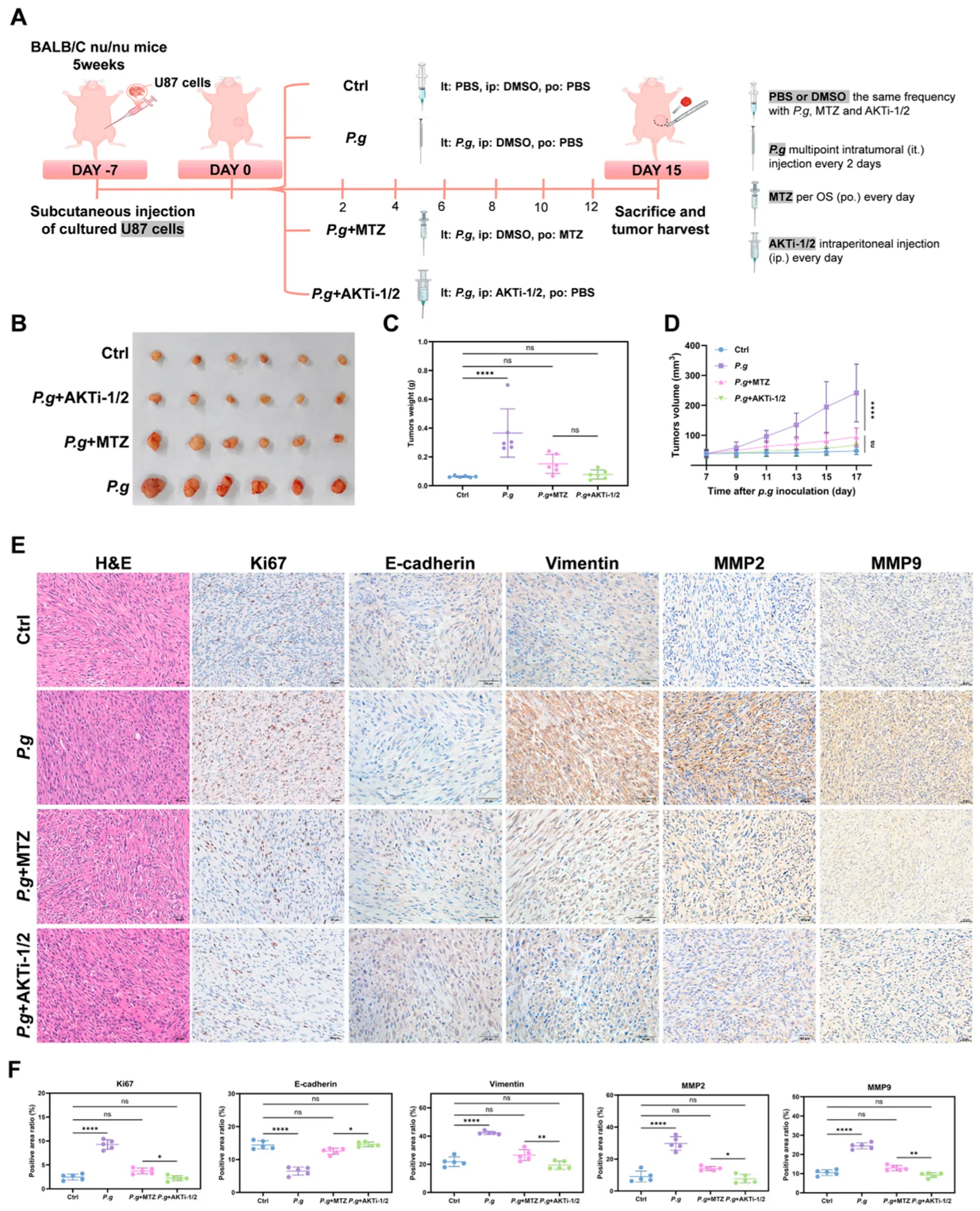
AKTi-1/2 and MTZ attenuate *P. gingivalis*-induced glioma progression in vivo. (**A**) Schematic diagram of generating subcutaneous glioma xenograft mouse model, the mice receiving intratumoral injections of PBS, *P. gingivalis*, *P. gingivalis* along with MTZ treatment and *P. gingivalis* along with AKTi-1/2 treatment, and tumor harvested. (**B**) Images of tumors taken from the tumor-bearing mice. (**C**) Tumor growth curves for the four groups. Tumor volume was measured at the indicated time points. Data were presented as mean ± SD (n = 6). (**D**) Quantification of final tumor weights. Data were presented as mean ± SD (n = 6). (**E**) Typical images of mouse tumor tissues stained with H&E and representative IHC staining images of tumor sections. Sections were stained with H&E or antibodies against Ki67, E-cadherin, Vimentin, MMP2, and MMP9. Scale bar: 50 μm. (**F**) Quantitative analysis of IHC staining. The positive staining area for Ki67, E-cadherin, Vimentin, MMP2, and MMP9 was quantified and normalized. Data were presented as mean ± SD (n = 6). Statistical analyses were performed using two-way ANOVA followed by Šídák’s multiple comparisons test for (**C**,**D**,**F**). Statistical significance: * *p* < 0.05, ** *p* < 0.01, **** *p* < 0.0001; ns, not significant. Exact *p* values are provided in Supplementary Table S1.

Our in vitro findings demonstrated that AKTi-1/2 effectively attenuated *P. gingivalis*-induced proliferation, migration, and invasion in GBM cells. To further extend these observations in vivo, we established a *P. gingivalis*-infected subcutaneous xenograft mouse model and systematically evaluated the therapeutic efficacy of AKTi-1/2, as well as the effects of MTZ on *P. gingivalis*-driven glioma malignant progression (Figure 8A).

Compared with the *P. gingivalis* group, both AKTi-1/2 and MTZ treatment remarkably reduced tumor volume and weight (Figure 8B–D). IHC analysis further revealed that AKTi-1/2 inhibited the *P. gingivalis*-induced upregulation of Ki67, Vimentin, MMP2, and MMP9, while restoring E-cadherin expression (Figure 8E,F). Similarly, MTZ intervention exhibited consistent protective effects by suppressing *P. gingivalis*-induced malignant phenotypes and abnormal expression of proliferation- and invasion-related markers (Figure 8B–F). Although AKTi-1/2 produced more pronounced changes in the assessed molecular markers than MTZ, the differences in tumor volume and weight between the two treatment groups were not statistically significant (Figure 8B–F). These findings indicate that both interventions attenuated tumor growth, while AKTi-1/2 showed stronger effects on proliferation-, mesenchymal transition-, and ECM remodeling-related markers.

## 4. Discussion

The oral-brain axis has emerged as an area of growing interest, yet the potential contribution of oral pathogens to glioma progression remains largely unexplored. In this study, we detected gingipain in 11 of 13 human glioma specimens and *P. gingivalis*-targeted 16S rRNA signals in 3 of 13 specimens. In cellular and animal models, *P. gingivalis* promotes glioma malignant progression by promoting proliferation, as well as migration and invasion through ECM remodeling and mesenchymal transition induction. Mechanistically, these tumor-promoting effects are mediated by activation of the PI3K/AKT/mTOR pathway. Crucially, pharmacological inhibition of AKT or elimination of *P. gingivalis* effectively attenuated these tumor-promoting effects. Together, these results suggest a potential association between *P. gingivalis* and glioma progression that warrants further investigation.

The blood–brain barrier (BBB) maintains central nervous system homeostasis but may become structurally and functionally impaired in pathological conditions, including GBM and AD [39,40,41]. Such compromise allows toxic factors or metabolites derived from gut and oral microbiota to traverse the BBB and enter the disease microenvironment, thereby accelerating disease progression and worsening clinical outcomes [42]. Previous studies have linked gut and oral microbiota to neurological disorders and brain tumors [17,43,44,45,46,47,48,49]. Periodontal pathogens or their products may migrate from the leaky gums to the brain via bloodstream or neuronal pathway [18,50,51]. Indeed, bacterial signatures overlapping with oral microbial communities have been reported in brain tumors [29]. Gingipains have also been detected in GBM tissues [30]. However, it remains unclear whether *P. gingivalis* itself exists in gliomas and what its possible role might be. In this study, IHC and FISH provide complementary evidence by detecting gingipain protein and *P. gingivalis*-targeted 16S rRNA, respectively, supporting the presence of *P. gingivalis*-associated signals in the human glioma tissues. However, these findings do not establish bacterial viability or persistent intratumoral colonization. The limited sample size and lack of non-tumor brain tissue controls also preclude determining whether these signals are only enriched in glioma. Larger independent cohorts with appropriate tissue controls and complementary detection methods are therefore needed to clarify the prevalence and biological significance of *P. gingivalis* in human gliomas.

*P. gingivalis* has been correlated with several malignancies, including oral squamous cell carcinomas [52], esophageal cancer [14], colorectal cancer [53], and pancreatic cancer [54]. Proposed mechanisms include modulation of oncogenic signaling, mesenchymal transition-related changes, suppression of apoptosis, and promotion of a proinflammatory tumor microenvironment [13,35,55]. Consistent with these reports, the present study revealed that *P. gingivalis* invaded GBM cells. Furthermore, once internalized, the intracellular *P. gingivalis* upregulated the expression of proliferation-related markers such as Ki-67, promote cell cycle progression toward the S phase, accelerates mitosis, and ultimately facilitates cell proliferation. In addition, this bacterium induces robust secretion of MMP2 and MMP9, which mediate ECM degradation and remodeling to form a tumor microenvironment conductive to GBM cells migration. Meanwhile, *P. gingivalis* downregulates the epithelial marker E-cadherin and upregulates the mesenchymal marker Vimentin, thereby triggering mesenchymal transition and markedly potentiating the invasive capacity of GBM cells.

The PI3K/AKT/mTOR signaling axis is involved in the regulation of cell survival, proliferation, migration, and invasion in GBM [3,56]. Our previous study showed that *P. gingivalis*-derived LPS activates this pathway [31]. Consistently, transcriptomic and Western blot analyses supported that *P. gingivalis* activates PI3K/AKT/mTOR signaling of GBM cells. This activation drives GBM cell proliferation and invasive properties (indicated by mesenchymal transition and ECM-remodeling). Pharmacological inhibition of AKT with AKTi-1/2 effectively attenuated malignant phenotypes in GBM, highlighting the essential role of this pathway in mediating the tumor-promoting functions of *P. gingivalis*. Both AKTi-1/2 and MTZ attenuated tumor growth in the animal model, although no significant differences in tumor volume or weight were observed between the two treatment groups.

This study has several limitations. First, although gingipain and *P. gingivalis*-targeted 16S rRNA signals were detected in some human glioma specimens, further studies are needed to clarify the viability of the bacteria and the route by which oral pathogens might reach glioma tissue. Second, in our in vivo study, we established the infected tumor model by directly injecting *P. gingivalis* into the tumors of subcutaneous xenografts. However, this approach has inherent limitations. On the one hand, it bypasses the natural physiological barriers; therefore, this model cannot demonstrate that naturally occurring oral *P. gingivalis* does reach intracranial gliomas, and future studies using orthotopic or systemic infection models are warranted to investigate this natural dissemination. On the other hand, the subcutaneous xenograft model in immunodeficient mice does not recapitulate the intracranial tumor microenvironment and lacks a functional immune system, thereby precluding a comprehensive evaluation of host immune responses. Given the immunosuppressive nature of glioma [57] and evidence that *P. gingivalis* can modulate anti-tumor immunity in other cancers [16,58,59], future studies using intracranial models and immunocompetent systems where appropriate may help clarify its potential effects on the glioma microenvironment. Further studies in this field will deepen our understanding of the intricate host–microbiota–immune crosstalk in glioma and facilitate the development of novel microbiota-targeted therapeutic strategies for glioma treatment.

## 5. Conclusions

In this study, gingipain was detected in 11 of 13 human glioma specimens, while *P. gingivalis*-targeted 16S rRNA were detected in 3 specimens, providing complementary evidence of *P. gingivalis*-associated signals in gliomas. In vivo and in vitro, *P. gingivalis* invaded GBM cells and promoted GBM cell proliferation, migration, and invasion, accompanied by mesenchymal transition-related changes and ECM remodeling. Our findings support the contribution of AKT signaling to these tumor-promoting effects. Together, these results suggest a potential association between *P. gingivalis* and glioma progression, warranting further investigation into its biological significance and the therapeutic potential of targeting host signaling pathways.

## Figures and Tables

**Table 1:** Demographic and clinicopathological information of the enrolled 13 patients.

Case No.	IHC-Gingipain *	FISH *	Age (Year)/Sex	Diagnosis (WHO CNS5)	WHO Grade	MGMT	1p/19q	+7/−10
1	+	−	53/F	Oligodendroglioma, IDH-mutant and 1p/19q-codeleted	III	Methylated	Codeleted	−
2	+	−	44/F	GBM, IDH-wildtype	IV	Methylated	Non-codeleted	−
3	+	−	43/F	GBM, IDH-wildtype	IV	Unmethylated	Non-codeleted	−
4	+	−	66/F	GBM, IDH-wildtype	IV	Unmethylated	Non-codeleted	+
5	+	+	60/M	GBM, IDH-wildtype	IV	Methylated	Non-codeleted	+
6	+	+	52/M	Astrocytoma, IDH-mutant	IV	Methylated	Non-codeleted	−
7	+	−	62/F	GBM, IDH-wildtype	IV	Methylated	Non-codeleted	−
8	+	+	65/M	GBM, IDH-wildtype	IV	Unmethylated	Non-codeleted	−
9	+	−	62/F	GBM, IDH-wildtype	IV	Unmethylated	Non-codeleted	−
10	+	−	39/F	Astrocytoma, IDH-mutant	II	Methylated	Non-codeleted	−
11	+	−	17/M	Diffuse hemispheric glioma, H3 G34-mutant	IV	Methylated	Non-codeleted	−
12	−	−	42/M	GBM, IDH-wildtype	IV	Unmethylated	Non-codeleted	+
13	−	−	62/M	GBM, IDH-wildtype	IV	Unmethylated	Non-codeleted	−

Abbreviations: IHC, immunohistochemistry; FISH, fluorescence in situ hybridization; IDH, Isocitrate dehydrogenase; MGMT, O6-methylguanine-DNA methyltransferase; GBM, glioblastoma; WHO, World Health Organization; CNS, central nervous system; F, female; M, male. * Note: +, positive; −, negative. For IHC and FISH, “+” indicates the positive detection of gingipain and *P. gingivalis* 16S rRNA signals, respectively.

**Table 2:** The primers used in qRT-PCR.

Gene	Primer Sequence (5′→3′)
Human Ki67	F: GAAAGAGTGGCAACCTGCCTTC R: GCACCAAGTTTTACTACATCTGCC
Human MMP2	F: AGCGAGTGGATGCCGCCTTTAA R: CATTCCAGGCATCTGCGATGAG
Human MMP9	F: GCCACTACTGTGCCTTTGAGTC R: CCCTCAGAGAATCGCCAGTACT
Human E-cadherin	F: GCCTCCTGAAAAGAGAGTGGAAG R: TGGCAGTGTCTCTCCAAATCCG
Human Vimentin	F: AGGCAAAGCAGGAGTCCACTGA R: ATCTGGCGTTCCAGGGACTCAT
Human GADPH	F: GTCTCCTCTGACTTCAACAGCG R: ACCACCCTGTTGCTGTAGCCAA

**Table 3:** The Primary antibodies used in Western blotting.

Name	Manufacture	Dilution Ratio
Anti-β-ACTIN	Abclonal, Wuhan, China	1:10,000
Anti-E-cadherin	HUABIO, Hangzhou, China	1:2000
Anti-MMP2	Abmart, Shanghai, China	1:1000
Anti-MMP9	HUABIO, Hangzhou, China	1:2000
Anti-Vimentin	Santa Cruz Biotechnology, Dallas, TX, USA	1:1000
Anti-*p*-PI3K	Abmart, Shanghai, China	1:2000
Anti-PI3K	Abmart, Shanghai, China	1:2000
Anti-*p*-AKT	Vazyme, Nanjing, China	1:2000
Anti-AKT	Vazyme, Nanjing, China	1:2000
Anti-*p*-mTOR	Abmart, Shanghai, China	1:1000
Anti-mTOR	Abmart, Shanghai, China	1:1000

## Data Availability

All data and materials included in this study are available upon request by contact with the corresponding author.

## Supplementary Materials

The following supporting information can be downloaded at https://www.mdpi.com/article/10.3390/cells15211998/s1, Figure S1: *P. gingivalis* activates the PI3K/AKT/mTOR signaling pathway in GBM cells. Table S1. Statistical tests and exact *p* values for comparisons presented in the figures.

## Abbreviations

AKTi	AKT inhibitor
CFSE	Carboxyfluorescein succinimidyl amino ester
DMEM	Dulbecco’s modified Eagle’s medium
DMSO	Dimethyl sulfoxide
EMT	Epithelial–Mesenchymal Transition
FISH	Fluorescence in situ hybridization
GBM	glioblastoma multiforme
GO	Gene ontology
H&E	Hematoxylin and eosin staining
IF	Immunofluorescence
IHC	Immunohistochemistry
KEGG	Kyoto Encyclopedia of Genes and Genomes
MOI	Multiplicity of infection
MTZ	Metronidazole
NS	No significance
*P. gingivalis*/*P.g*	*Porphyromonas gingivalis*
qRT-PCR	quantitative real-time reverse transcription polymerase chain reaction
TEM	Transmission electron microscope
WB	Western blot
