# Supplementary material for: Porphyromonas gingivalis Promotes Glioma Progression via the PI3K/AKT/mTOR Signaling Pathway
Source: Cells. 2026 Oct 31;15(21):1998. doi: 10.3390/cells15211998 (PMC13762273; doi:10.3390/cells15211998)
Supplement: Supplementary file 1 [file cells-15-01998-s001.zip › Supplementary File/Table S1. Statistical tests and exact p values for comparisons presented in the figures.pdf]

Figure/panel	Cell line/group	Comparison	Statistical test	Excat *p* value
Fig.2A	U87	24h, ctrl vs. *P.g*	two-way ANOVA followed by Šídák's multiple comparisons test	0.0015
	U87	48h, ctrl vs. *P.g*		0.0004
	U87	72h, ctrl vs. *P.g*		< 0.0001
	U251	24h, ctrl vs. *P.g*		0.0112
	U251	48h, ctrl vs. *P.g*		0.0243
	U251	72h, ctrl vs. *P.g*		< 0.0001
Fig.2C	U87	ctrl vs. *P.g*	unpaired two-tailed Student's t-test	0.0478
	U251	ctrl vs. *P.g*		0.0499
Fig.2D	U87	ctrl vs. *P.g*	unpaired two-tailed Student's t-test	0.0196
	U251	ctrl vs. *P.g*		0.0003
Fig.2G	U87	G0/G1, ctrl vs. *P.g*	two-way ANOVA followed by Šídák's multiple comparisons test	0.0082
	U87	S, ctrl vs. *P.g*		0.0130
	U87	G2/M, ctrl vs. *P.g*		0.9926
	U251	G0/G1, ctrl vs. *P.g*		0.0019
	U251	S, ctrl vs. *P.g*		0.9849
	U251	G2/M, ctrl vs. *P.g*		0.0033
Fig.2H	U87	ctrl vs. *P.g*	unpaired two-tailed Student's t-test	0.0481
	U251	ctrl vs. *P.g*		0.0286
Fig.2J	U87	ctrl vs. *P.g*	unpaired two-tailed Student's t-test	0.0128
	U251	ctrl vs. *P.g*		0.0064
Fig.2A	U87	24h, ctrl vs. *P.g*	two-way ANOVA followed by Šídák's multiple comparisons test	0.0149
	U87	48h, ctrl vs. *P.g*		0.0344
	U87	72h, ctrl vs. *P.g*		0.0036
	U251	24h, ctrl vs. *P.g*		0.0003
	U251	48h, ctrl vs. *P.g*		0.0164
	U251	72h, ctrl vs. *P.g*		0.0383
Fig.2D	U87 Migration	ctrl vs. *P.g*	two-way ANOVA followed by Šídák's multiple comparisons test	< 0.0001
	U251 Migration	ctrl vs. *P.g*		0.0065
Fig.2D	U87 Invasion	ctrl vs. *P.g*		0.0065
	U251 Invasion	ctrl vs. *P.g*		0.0413
Fig.4C	U87 E-cadherin	ctrl vs. *P.g*	two-way ANOVA	0.0491
	U87 Vimentin	ctrl vs. *P.g*		0.0015

	U87 MMP2	ctrl vs. *P.g*	followed by Šídák's multiple comparisons test	0.0110
	U87 MMP9	ctrl vs. *P.g*		0.0281
	U251 E-cadherin	ctrl vs. *P.g*		0.0180
	U251 Vimentin	ctrl vs. *P.g*		0.0280
	U251 MMP2	ctrl vs. *P.g*		0.0002
	U251 MMP9	ctrl vs. *P.g*		<0.0001
Fig.4E	U87 E-cadherin	ctrl vs. *P.g*	two-way ANOVA followed by Šídák's multiple comparisons test	0.0001
	U87 Vimentin	ctrl vs. *P.g*		0.009
	U87 MMP2	ctrl vs. *P.g*		0.0002
	U87 MMP9	ctrl vs. *P.g*		< 0.0001
	U251 E-cadherin	ctrl vs. *P.g*		0.008
	U251 Vimentin	ctrl vs. *P.g*		< 0.0001
	U251 MMP2	ctrl vs. *P.g*		0.0002
	U251 MMP9	ctrl vs. *P.g*		0.0266
Fig.5C		ctrl vs. *P.g*	unpaired two-tailed Student's t-test	0.0057
Fig.5D		ctrl vs. *P.g*		< 0.0001
Fig.5F		ctrl vs. *P.g*	unpaired two-tailed Student's t-test	< 0.0001
Fig.5H	E-cadherin	ctrl vs. *P.g*	unpaired two-tailed Student's t-test	< 0.0001
	Vimentin	ctrl vs. *P.g*		< 0.0001
	MMP2	ctrl vs. *P.g*		< 0.0001
	MMP9	ctrl vs. *P.g*		< 0.0001
Fig.6B	U87 p-AKT/AKT	ctrl vs. *P.g*	two-way ANOVA followed by Šídák's multiple comparisons test	0.0002
		ctrl vs. *P.g*+AKTi-1/2		< 0.0001
		ctrl vs. AKTi-1/2		< 0.0001
		P.g vs. *P.g*+AKTi-1/2		< 0.0001
		P.g+AKTi-1/2 vs. AKTi-1/2		0.5091
	U87 p-mTOR/mTOR	ctrl vs. *P.g*		< 0.0001
		ctrl vs. *P.g*+AKTi-1/2		0.0266
		ctrl vs. AKTi-1/2		0.0120
		P.g vs. *P.g*+AKTi-1/2		< 0.0001
		P.g+AKTi-1/2		0.9994

		vs. AKTi-1/2		
	U251 p-AKT/AKT	ctrl vs. *P.g*	two-way ANOVA followed by Šídák's multiple comparisons test	< 0.0001
		ctrl vs. *P.g*+AKTi-1/2		< 0.0001
		ctrl vs. AKTi-1/2		< 0.0001
		P.g vs. *P.g*+AKTi-1/2		< 0.0001
		P.g+AKTi-1/2 vs. AKTi-1/2		0.9435
	U251 p-mTOR/mTOR	ctrl vs. *P.g*		0.0312
		ctrl vs. *P.g*+AKTi-1/2		0.0212
		ctrl vs. AKTi-1/2		0.009
		P.g vs. *P.g*+AKTi-1/2		< 0.0001
		P.g+AKTi-1/2 vs. AKTi-1/2		0.9736
Fig.6C	U87 24h	ctrl vs. *P.g*	Two-way ANOVA followed by Tukey's Honestly Significant Difference test	<0.0001
		ctrl vs. *P.g*+AKTi-1/2		0.3455
		ctrl vs. AKTi-1/2		0.0312
		P.g vs. *P.g*+AKTi-1/2		<0.0001
		P.g+AKTi-1/2 vs. AKTi-1/2		0.5869
	U87 48h	ctrl vs. *P.g*		<0.0001
		ctrl vs. *P.g*+AKTi-1/2		0.1365
		ctrl vs. AKTi-1/2		0.0442
		P.g vs. *P.g*+AKTi-1/2		<0.0001
		P.g+AKTi-1/2 vs. AKTi-1/2		0.9435
	U87 72h	ctrl vs. *P.g*		<0.0001
		ctrl vs. *P.g*+AKTi-1/2		0.8428
		ctrl vs. AKTi-1/2		0.0482
		P.g vs.		<0.0001

		P.g+AKTi-1/2		
		P.g+AKTi-1/2 vs. AKTi-1/2		0.2337
	U251 24h	ctrl vs. *P.g*		0.0173
		ctrl vs. *P.g*+AKTi-1/2		0.1426
		ctrl vs. AKTi-1/2		0.0060
		P.g vs. *P.g*+AKTi-1/2		<0.0001
		P.g+AKTi-1/2 vs. AKTi-1/2		0.4808
	U251 48h	ctrl vs. *P.g*		0.0088
		ctrl vs. *P.g*+AKTi-1/2		0.0862
		ctrl vs. AKTi-1/2		0.0046
		P.g vs. *P.g*+AKTi-1/2		<0.0001
		P.g+AKTi-1/2 vs. AKTi-1/2		0.5745
	U251 72h	ctrl vs. *P.g*		0.0033
		ctrl vs. *P.g*+AKTi-1/2		0.4070
		ctrl vs. AKTi-1/2		0.0145
		P.g vs. *P.g*+AKTi-1/2		<0.0001
		P.g+AKTi-1/2 vs. AKTi-1/2		0.3307
Fig.7D	U87 migration	ctrl vs. *P.g*	Two-way ANOVA followed by Tukey's Honestly Significant Difference test	<0.0001
		ctrl vs. *P.g*+AKTi-1/2		0.1323
		ctrl vs. AKTi-1/2		0.0005
		P.g vs. *P.g*+AKTi-1/2		<0.0001
		P.g+AKTi-1/2 vs. AKTi-1/2		0.0539
	U251 migration	ctrl vs. *P.g*		<0.0001
		ctrl vs. *P.g*+AKTi-1/2		0.9051
		ctrl vs. AKTi-		0.0297

		1/2		
		P.g vs. *P.g*+AKTi-1/2		<0.0001
		P.g+AKTi-1/2 vs. AKTi-1/2		0.1066
	U87 invasion	ctrl vs. *P.g*		<0.0001
		ctrl vs. *P.g*+AKTi-1/2		0.2442
		ctrl vs. AKTi-1/2		0.0015
		P.g vs. *P.g*+AKTi-1/2		<0.0001
		P.g+AKTi-1/2 vs. AKTi-1/2		0.0746
	U251 invasion	ctrl vs. *P.g*		<0.0001
		ctrl vs. *P.g*+AKTi-1/2		0.4302
		ctrl vs. AKTi-1/2		0.0155
		P.g vs. *P.g*+AKTi-1/2		<0.0001
		P.g+AKTi-1/2 vs. AKTi-1/2		0.2666
Fig.7B	U87 24h	ctrl vs. *P.g*	Two-way ANOVA followed by Tukey's Honestly Significant Difference test	0.0019
		ctrl vs. *P.g*+AKTi-1/2		0.9903
		ctrl vs. AKTi-1/2		0.0281
		P.g vs. *P.g*+AKTi-1/2		0.0010
		P.g+AKTi-1/2 vs. AKTi-1/2		0.0505
	U87 48h	ctrl vs. *P.g*		0.0438
		ctrl vs. *P.g*+AKTi-1/2		0.7219
		ctrl vs. AKTi-1/2		0.0272
		P.g vs. *P.g*+AKTi-1/2		0.0053
		P.g+AKTi-1/2 vs. AKTi-1/2		0.1887
	U251 24h	ctrl vs. *P.g*		0.0006
		ctrl vs.		0.9837

		P.g+AKTi-1/2		
		ctrl vs. AKTi-1/2		0.0417
		P.g vs. *P.g*+AKTi-1/2		0.0003
		P.g+AKTi-1/2 vs. AKTi-1/2		0.0822
	U251 48h	ctrl vs. *P.g*		<0.0001
		ctrl vs. *P.g*+AKTi-1/2		0.0895
		ctrl vs. AKTi-1/2		0.0042
		P.g vs. *P.g*+AKTi-1/2		<0.0001
		P.g+AKTi-1/2 vs. AKTi-1/2		0.4325
Fig.7E	U87 E-cadherin	ctrl vs. *P.g*	Two-way ANOVA followed by Tukey's Honestly Significant Difference test	0.0015
		ctrl vs. *P.g*+AKTi-1/2		0.9979
		ctrl vs. AKTi-1/2		0.0356
		P.g vs. *P.g*+AKTi-1/2		0.0009
		P.g+AKTi-1/2 vs. AKTi-1/2		0.0536
	U87 Vimentin	ctrl vs. *P.g*		0.0020
		ctrl vs. *P.g*+AKTi-1/2		0.9925
		ctrl vs. AKTi-1/2		0.0352
		P.g vs. *P.g*+AKTi-1/2		0.0009
		P.g+AKTi-1/2 vs. AKTi-1/2		0.0657
	U87 MMP2	ctrl vs. *P.g*		0.0002
		ctrl vs. *P.g*+AKTi-1/2		0.3659
		ctrl vs. AKTi-1/2		0.0365
		P.g vs. *P.g*+AKTi-1/2		<0.0001
		P.g+AKTi-1/2 vs. AKTi-1/2		0.6336

	U87 MMP9	ctrl vs. *P.g*		<0.0001
		ctrl vs. *P.g*+AKTi-1/2		0.1495
		ctrl vs. AKTi-1/2		0.0172
		P.g vs. *P.g*+AKTi-1/2		<0.0001
		P.g+AKTi-1/2 vs. AKTi-1/2		0.7635
Fig.7E	U251 E-cadherin	ctrl vs. *P.g*	Two-way ANOVA followed by Tukey's Honestly Significant Difference test	0.0098
		ctrl vs. *P.g*+AKTi-1/2		>0.9999
		ctrl vs. AKTi-1/2		0.0442
		P.g vs. *P.g*+AKTi-1/2		0.0084
		P.g+AKTi-1/2 vs. AKTi-1/2		0.0506
	U251 Vimentin	ctrl vs. *P.g*		0.0110
		ctrl vs. *P.g*+AKTi-1/2		0.3374
		ctrl vs. AKTi-1/2		0.0479
		P.g vs. *P.g*+AKTi-1/2		<0.0001
		P.g+AKTi-1/2 vs. AKTi-1/2		0.7376
	U251 MMP2	ctrl vs. *P.g*		0.0001
		ctrl vs. *P.g*+AKTi-1/2		0.8908
		ctrl vs. AKTi-1/2		0.0485
		P.g vs. *P.g*+AKTi-1/2		<0.0001
		P.g+AKTi-1/2 vs. AKTi-1/2		0.2058
	U251 MMP9	ctrl vs. *P.g*		<0.0001
		ctrl vs. *P.g*+AKTi-1/2		0.6459
		ctrl vs. AKTi-1/2		0.0350
		P.g vs. *P.g*+AKTi-1/2		<0.0001

		P.g+AKTi-1/2 vs. AKTi-1/2		0.3458
Fig.7G	U87 E-cadherin	ctrl vs. *P.g*	Two-way ANOVA followed by Tukey's Honestly Significant Difference test	0.0316
		ctrl vs. *P.g*+AKTi-1/2		0.0797
		ctrl vs. AKTi-1/2		0.0408
		P.g vs. *P.g*+AKTi-1/2		<0.0001
		P.g+AKTi-1/2 vs. AKTi-1/2		0.9902
	U87 Vimentin	ctrl vs. *P.g*		<0.0001
		ctrl vs. *P.g*+AKTi-1/2		0.5127
		ctrl vs. AKTi-1/2		0.0407
		P.g vs. *P.g*+AKTi-1/2		<0.0001
		P.g+AKTi-1/2 vs. AKTi-1/2		0.5042
	U87 MMP2	ctrl vs. *P.g*		0.0063
		ctrl vs. *P.g*+AKTi-1/2		0.2975
		ctrl vs. AKTi-1/2		0.0482
		P.g vs. *P.g*+AKTi-1/2		<0.0001
		P.g+AKTi-1/2 vs. AKTi-1/2		0.7853
	U87 MMP9	ctrl vs. *P.g*		0.0123
		ctrl vs. *P.g*+AKTi-1/2		0.0603
		ctrl vs. AKTi-1/2		0.0439
		P.g vs. *P.g*+AKTi-1/2		<0.0001
		P.g+AKTi-1/2 vs. AKTi-1/2		0.9990
Fig.7G	U251 E-cadherin	ctrl vs. *P.g*	Two-way ANOVA followed by Tukey's Honestly	<0.0001
		ctrl vs. *P.g*+AKTi-1/2		0.8590
		ctrl vs. AKTi-1/2		0.0073

		P.g vs. *P.g*+AKTi-1/2	Significant Difference test	<0.0001
		P.g+AKTi-1/2 vs. AKTi-1/2		0.0501
	U251 Vimentin	ctrl vs. *P.g*		0.0087
		ctrl vs. *P.g*+AKTi-1/2		0.1290
		ctrl vs. AKTi-1/2		0.0195
		P.g vs. *P.g*+AKTi-1/2		<0.0001
		P.g+AKTi-1/2 vs. AKTi-1/2		0.8312
	U251 MMP2	ctrl vs. *P.g*		0.0027
		ctrl vs. *P.g*+AKTi-1/2		0.3857
		ctrl vs. AKTi-1/2		0.0023
		P.g vs. *P.g*+AKTi-1/2		<0.0001
		P.g+AKTi-1/2 vs. AKTi-1/2		0.1169
	U251 MMP9	ctrl vs. *P.g*		<0.0001
		ctrl vs. *P.g*+AKTi-1/2		0.1372
		ctrl vs. AKTi-1/2		0.0394
		P.g vs. *P.g*+AKTi-1/2		<0.0001
		P.g+AKTi-1/2 vs. AKTi-1/2		0.9360
Fig.8C		ctrl vs. *P.g*	Two-way ANOVA followed by Tukey's Honestly Significant Difference test	<0.0001
		ctrl vs. *P.g*+MTZ		0.3611
		ctrl vs. *P.g*+AKTi-1/2		0.9935
		P.g+MTZ vs. *P.g*+AKTi-1/2		0.5049
Fig.8D		ctrl vs. *P.g*	Two-way ANOVA followed by Tukey's Honestly	<0.0001
		ctrl vs. *P.g*+MTZ		0.0587
		ctrl vs. *P.g*+AKTi-1/2		0.7304

		P.g+MTZ vs. *P.g*+AKTi-1/2	Significant Difference test	0.4356
Fig.8F	Ki67	ctrl vs. *P.g*	Two-way ANOVA followed by Tukey's Honestly Significant Difference test	<0.0001
		ctrl vs. *P.g*+MTZ		0.0547
		ctrl vs. *P.g*+AKTi-1/2		0.9373
		P.g+MTZ vs. *P.g*+AKTi-1/2		0.0176
	E-cadherin	ctrl vs. *P.g*	Two-way ANOVA followed by Tukey's Honestly Significant Difference test	<0.0001
		ctrl vs. *P.g*+MTZ		0.0528
		ctrl vs. *P.g*+AKTi-1/2		0.9942
		P.g+MTZ vs. *P.g*+AKTi-1/2		0.0325
	Vimentin	ctrl vs. *P.g*	Two-way ANOVA followed by Tukey's Honestly Significant Difference test	<0.0001
		ctrl vs. *P.g*+MTZ		0.1099
		ctrl vs. *P.g*+AKTi-1/2		0.5850
		P.g+MTZ vs. *P.g*+AKTi-1/2		0.0090
	MMP2	ctrl vs. *P.g*	Two-way ANOVA followed by Tukey's Honestly Significant Difference test	<0.0001
		ctrl vs. *P.g*+MTZ		0.0864
		ctrl vs. *P.g*+AKTi-1/2		0.9050
		P.g+MTZ vs. *P.g*+AKTi-1/2		0.0236
	MMP9	ctrl vs. *P.g*	Two-way ANOVA followed by Tukey's Honestly Significant Difference test	<0.0001
		ctrl vs. *P.g*+MTZ		0.0825
		ctrl vs. *P.g*+AKTi-1/2		0.5059
		P.g+MTZ vs. *P.g*+AKTi-1/2		0.0050
Fig.S1B	U87	*p*-PI3K/PI3K	two-way ANOVA followed by Šídák's multiple comparisons test	<0.0001
		p-AKT/AKT		0.0442
		p-MTOR/MTOR		0.0019

		p-PI3K/PI3K	two-way ANOVA followed by Šídák's multiple comparisons test	0.0468
		p-AKT/AKT		0.0068
	U251	*p*-MTOR/MTOR		0.0003
